# Mortality and Readmission Rates Among Patients With COVID-19 After Discharge From Acute Care Setting With Supplemental Oxygen

**DOI:** 10.1001/jamanetworkopen.2021.3990

**Published:** 2021-04-01

**Authors:** Josh Banerjee, Catherine P. Canamar, Christian Voyageur, Soodtida Tangpraphaphorn, Anabel Lemus, Charles Coffey, Noah Wald-Dickler, Paul Holtom, Jan Shoenberger, Michael Bowdish, Hal F. Yee, Brad Spellberg

**Affiliations:** 1 Los Angeles County + University of Southern California (LAC+USC) Medical Center, Los Angeles; 2 Department of Emergency Medicine, Keck School of Medicine at University of Southern California, Los Angeles; 3 Department of Surgery, Keck School of Medicine at University of Southern California, Los Angeles; 4 Los Angeles County Department of Health Services, Los Angeles, California

## Abstract

**Importance:**

To optimize patient outcomes and preserve critical acute care access during the COVID-19 pandemic, the Los Angeles County Department of Health Services developed the SAFE @ HOME O_2_ Expected Practice (expected practice), enabling ambulatory oxygen management for COVID-19.

**Objective:**

To assess outcomes of patients with COVID-19 pneumonia discharged via the expected practice approach to home or quarantine housing with supplemental home oxygen.

**Design, Setting, and Participants:**

This retrospective cohort study included 621 adult patients with COVID-19 pneumonia who were discharged from 2 large urban public hospitals caring primarily for patients receiving Medicaid from March 20 to August 19, 2020. Patients were included in the analysis cohort if they received emergency or inpatient care for COVID-19 and were discharged with home oxygen.

**Interventions:**

Patients receiving at least 3 L per minute of oxygen, stable without other indication for inpatient care, were discharged from either emergency or inpatient encounters with home oxygen equipment, educational resources, and nursing telephone follow-up within 12 to 18 hours of discharge. Nurses provided continued telephone follow up as indicated, always with physician back-up.

**Main Outcomes and Measures:**

All-cause mortality and all-cause 30-day return admission.

**Results:**

A total of 621 patients with COVID-19 pneumonia (404 male [65.1%] and 217 female [34.9%]) were discharged with home oxygen. Median age of these patients was 51 years (interquartile range, 45-61 years), with 149 (24.0%) discharged from the emergency department and 472 (76%) discharged from inpatient encounters. The all-cause mortality rate was 1.3% (95% CI, 0.6%-2.5%) and the 30-day return hospital admission rate was 8.5% (95% CI, 6.2%-10.7%) with a median follow-up time of 26 days (interquartile range, 15-55 days). No deaths occurred in the ambulatory setting.

**Conclusions and Relevance:**

In this cohort study, patients with COVID-19 pneumonia discharged on home oxygen had low rates of mortality and return admission within 30 days of discharge. Ambulatory management of COVID-19 with home oxygen has an acceptable safety profile, and the expected practice approach may help optimize outcomes, by ensuring right care in the right place at the right time and preserving access to acute care during the COVID-19 pandemic.

## Key Points

**Question:**

What are the mortality and readmission rates in patients with COVID-19 pneumonia discharged according to an expected practice approach with supplemental home oxygen?

**Findings:**

In this cohort study of 621 patients with COVID-19 discharged with supplemental home oxygen from emergency department and inpatient encounters at 2 large urban medical centers, the all-cause mortality rate was 1.3% and the all-cause 30-day return hospital admission rate was 8.5%. No patients died in the ambulatory setting or in transit when returning to acute care setting.

**Meaning:**

In this study, a careful and systematic expected practice approach to treatment of patients with COVID-19 using home oxygen was associated with low all-cause mortality and low 30-day return admission rates.

## Teaser

This cohort study assesses mortality and readmission rates of adult patients with COVID-19 pneumonia discharged from 2 centers in Los Angeles County, California, via an expected practice approach to home or quarantine housing with supplemental home oxygen.

## Introduction

As presented by the Institute for Healthcare Innovation, a fundamental principle of high quality patient-centered care is to provide the right care in the right place at the right time.^1^ This practice enhances patient safety by transitioning patients to outpatient care as soon as there are no conditions that require acute inpatient care. The COVID-19 global pandemic has created challenges with respect to ensuring safety of inpatients^2,3^ and strain on acute care hospital capacity,^4,5^ creating a need for identifying innovative ways to ensure right care, right place, and right time.

As is the same for other health systems, the COVID-19 pandemic created concerns regarding patient safety and access for the Los Angeles County Department of Health Services (LA County DHS), which is the second largest municipal health system in the US. The LA County DHS has committed to reducing practice variation across all specialties, and an important tool in standardization is the expected practice (EP).^6^

The EPs are developed collaboratively by groups of primary and specialty care front-line physicians in a manner that is practical and implementable. In March 2020, recognizing the need to provide real-time decision support on a global pandemic for a workforce of approximately 22 000 employees, the LA County DHS created a new library of EPs specifically devoted to the evaluation and management of COVID-19. An important aspect of ensuring patient safety and maintaining hospital access during the pandemic was the establishment and implementation of an EP about use of home oxygen to enable earlier discharges.

## Methods

### Study Design and Setting

This multicentered retrospective cohort study included patients who were discharged to home or quarantine housing with supplemental home oxygen after receiving acute care for COVID-19, in accordance with the EP. This study followed the Strengthening the Reporting of Observational Studies in Epidemiology (STROBE) reporting guideline for cohort studies. This study was approved as expedited with a waiver of informed consent by the Health Sciences Institutional Review Board at the University of Southern California in accordance with 45 CFR §46. Patients were included if they tested positive for SARS-CoV-2 RNA on nasopharyngeal swab and received emergency or inpatient care for COVID-19 pneumonia at Los Angeles County + University of Southern California (LAC+USC) or Olive View + University of California at Los Angeles (Olive View UCLA) Medical Center, from March 20 to August 19, 2020.

Eligibility required ability to self-manage or manage with assistance of caretaker; access to secure housing, whether own or temporarily provided through Department of Public Health for an indefinite interval to conclude only after supportive oxygen was no longer necessary; and reliable telephone access. All patients discharged with home oxygen met EP criteria; patients not meeting criteria were not authorized for discharge with home oxygen.

Patients were followed up through September 30, 2020. Polymerase chain reaction tests used at the hospitals included the Xpert Xpress SARS-CoV-2 (Cepheid), the QiaSTAT-Dx Respiratory SARS-CoV (Qiagen), the Biofire COVID-19 test (Biofire Diagnostics), and send-out testing to a reference laboratory (Quest Diagnostics).

### The Expected Practice Approach

The COVID-19 Admission, Discharge, and Home O_2_ (SAFE @ HOME O_2_) EP (eAppendix 1 in the Supplement) included several principal care expectations (Table 1). The summary of patient selection and intervention steps for the EP are listed in the Box. First, patients with COVID-19 pneumonia who were clinically stable and requiring at least 3 L per minute of nasal cannula oxygen to achieve at least 92% oxygen saturation should be discharged and treated in an ambulatory setting with strict return precautions, in the absence of any other indication for acute care. Second, to ensure safe ambulatory management of COVID-19, DHS hospitals should establish local processes to ensure reliable availability of oxygen durable medical equipment (DME) delivery and vendor access after discharge. This availability included stocking portable oxygen and pulse oximeters from the DME vendor in the emergency department so patients could be sent home directly from the emergency department after hours, without waiting for the DME vendor’s normal business hours to arrive the next day. Third, patients discharged on oxygen must receive a nurse telephone call, with physician support if needed, within the first 12 to 18 hours after discharge.

**Table 1.** Characteristics of 621 Patients Enrolled

Characteristic	No. (%)
No.	621
Age, median (IQR), y	51 (45-61)
Sex	
Female	217 (34.9)
Male	404 (65.1)
Enrollment site	
LAC+USC Medical Center	506 (81.5)
Olive View Medical Center	115 (18.5)
Encounter setting at discharge	
Emergency department	149 (24.0)
Inpatient	472 (76.0)
O_2_ L/min, median (IQR)	2.0 (2.0-3.0)
Coexisting disorders	
Asthma	25 (4.0)
BMI ≥30 (obesity)	114 (18.4)
Cancer	17 (2.7)
Cerebral infarction	1 (0.2)
Cerebrovascular disease	2 (0.3)
Chronic kidney disease	41 (6.6)
Chronic liver disease	25 (4.0)
COPD	8 (1.3)
Coronary heart disease	17 (2.7)
Diabetes	235 (37.8)
End stage kidney disease	17 (2.7)
Heart failure	20 (3.2)
Hepatitis	3 (0.5)
Homelessness^a^	3 (0.5)
Hypertension	212 (34.1)
Immunodeficiency	1 (0.2)
Psychiatric disorder	33 (5.3)
Substance use disorder	33 (5.3)

Abbreviations: BMI, body mass index (calculated as weight in kilograms divided by height in meters squared); COPD, chronic obstructive pulmonary disease; IQR, interquartile range; LAC+USC, Los Angeles County + University of Southern California.

^a^ Patients who were homeless were discharged to public health housing for patients with positive test results for COVID-19.

Box. **Summary of Patient Selection and Intervention Steps for the SAFE @ HOME O_2_ Expected Practice**

**Patient Selection Criteria**

Improving clinical trajectory
Comfortable at rest and with minimal exertion (eg, able to get out of bed, ambulate to bathroom and back)
Stable heart rate (≤110 beats/min) and respiratory rate (≤22 breaths/min)
Stable oxygen saturation of at least 92%, with at least 3L/min of supplemental home oxygen
No other reasons for continued evaluation and management in acute care setting

**Interventions**

Patient receives educational handout and video prior to discharge
Facility-based team including RN and MD perform telephone follow-ups 7 days a week
First telephone contact occurs within 12 to 18 hours of discharge from the acute care setting
Regular short-term follow-up continues by telephone until no longer clinically necessary
Facility dispenses equipment (pulse oximeter, oxygen tank, concentrator), vendor also provides ongoing support

MD indicates doctor of medicine; RN, registered nurse.

The clinical follow-up program consisted of a nurse manager who assigned cases and performed quality assurance, 3 nurses who shared a schedule to perform follow-up calls, a physician on call for support, and the equipment vendor support team who were accessible at all times for DME troubleshooting. Calls were performed 7 days a week and continued daily until patients demonstrated, through verbal teach-back, that they understood how to use oxygen equipment and indications for return to acute care.

Patient education reinforced during these calls was consistent with that provided on discharge. Patients were given self-management instructions, including a weaning protocol. Respiratory therapists from the vendor also performed periodic checks on patients to help reinforce instructions. Collectively, the team determined appropriate timing of discontinuation of oxygen.

In addition to verbal education, at the time of discharge, patients received a printed educational handout (eAppendix 2 in the Supplement) and access to an educational video regarding safety parameters and self-management with home oxygen (Video). While telephone access was a requirement for discharge through the program, literacy or access to video playback device was not, although both were accounted for in discharge and follow-up education. Supplemental education materials were available in primary languages of English and Spanish, but both patients and staff had access to on-call translation services for other languages.

**Video.** Home Oxygen Instructions for Discharged Patients With COVID-19 This instructional video was one component of a program to maximize acute care beds for patients with COVID-19 requiring inpatient care by discharging otherwise clinically stable patients with low-level oxygen requirements who could safely manage the equipment to home or quarantine housing with proper supplies and follow-up.

### Data Sources and Measurement

The act of discharge on home oxygen triggered an automatic notice to the home follow-up nursing and physician EP team. The EP team maintained a contact list and call log of clinical support provided to patients during their participation. For the study cohort, demographic, clinical, and return admission data were obtained through automated query of the shared electronic health record. Principal diagnoses and deaths were confirmed through physician medical record review. For patients receiving primary or specialty care within the LA County DHS network, postdischarge outpatient encounters were also included as follow-up points. The EP team used the vendor report to validate that all patients with COVID-19 pneumonia receiving DME were included and followed up until no longer indicated clinically.

### Statistical Analysis

For primary analysis, we calculated summary descriptive statistics, median and interquartile range, for patients discharged while receiving home oxygen for COVID-19 pneumonia. Data were collected in MS Excel 2016 (Microsoft). Statistical testing was performed using SAS Enterprise Guide, version 7.1 (SAS Institute Inc).

## Results

During the study period a total of 621 unique patients with COVID-19 pneumonia, 217 (34.9%) female and 404 (65.1%) male, were discharged from an index acute care encounter with home oxygen. Of these patients, 149 (24.0%) were discharged from the emergency department and 472 (76.0%) were discharged from inpatient admissions (Table 1). The median age of the cohort was 51 years (interquartile range, 45-61 years), and consistent with the general population served by both hospitals, patients were primarily insured by Medicaid (76%) and Spanish-speaking (84%).

Over a median follow-up time of 26 days (interquartile range, 25-27 days), patients discharged receiving home oxygen for COVID-19 pneumonia were observed to have an all-cause mortality rate of 1.3% (95% CI, 0.6%-2.5%), and none of these patients died at home or during transport back to acute care (Table 2). In addition, while formal cohort analysis was not performed, hospital mortality for patients readmitted after trial of home oxygen (8 of 53 [15%]) was consistent with that of overall observed hospital mortality for patients admitted to study institutions for COVID-19 pneumonia who did not have a preceding or subsequent trial of home oxygen (147 of 1044 [14%]). No patients were identified as lost to follow-up by conclusion of study. The observed rate of all-cause return admission within 30 days was 8.5% (95% CI, 6.2%-10.7%).

**Table 2.** Outcomes in 621 Patients With COVID-19

Outcome	No. (%) [95% CI]
Follow-up time, median (IQR), d	26.0 (15-55) [24-30]
30-d Return hospital readmission, any facility	53 (8.5) [6.2-10.7]
Deaths, any facility^a^	8 (1.3) [0.6-2.5]

Abbreviation: IQR, interquartile range.

^a^ All deaths occurred on subsequent admission to inpatient setting, either because of progression of COVID-19, progression of other underlying disease, or both. No deaths occurred in the field or at home.

## Discussion

In this cohort study of 621 patients with COVID-19 pneumonia, disposition to ambulatory setting with low levels of supplementary oxygen was associated with low all-cause mortality and low 30-day return admission rates. The observed 30-day readmission rate in this study was below the nationally reported rates for Medicaid (13.7%) and consistent with privately insured (8.6%) patients.^7^ The observed 30-day readmission rate for these home oxygen patients was also lower than the overall post-acute care 30-day readmission rate for DHS patients (15.2%), as reported to California Department of Health Care Services in 2020. These outcomes underscore the safety of a carefully implemented home oxygen program for patients with COVID-19 pneumonia.

### Limitations

This study has limitations. The primary limitations of the study are its observational nature and potential for indication, selection, and spectrum biases in program enrollment. Comparable data on patients not discharged but not requiring home oxygen as well as acute care duration data were not available. All of these factors limit the generalizability of this study findings. Nevertheless, the low observed mortality rate is below the range reported in large surveillance studies of outpatients with COVID-19 (2%-4%).^8,9^ Furthermore, no patients died at home; all deaths after discharge occurred after subsequent readmission because of disease progression. Therefore, it is unlikely that a control group of patients who remained in the hospital rather than being discharged on home oxygen would have had a lower mortality rate.

## Conclusions

In this cohort study, ambulatory management of COVID-19 pneumonia with supplemental home oxygen, through a carefully devised and executed EP, was associated with low all-cause mortality and low 30-day return admission rates. This EP may be considered part of a strategy to ensure right care, right place, and right time for patients with COVID-19 pneumonia, and to preserve acute care access during the pandemic.

## Notes

Supplement. **eAppendix 1.** DHS Expected Practices: COVID19 Admission, Discharge, and Home O_2_ **eAppendix 2.** DHS Safer at Home – Home Oxygen Discharge Instructions
