# Supplementary material for: Mortality and Readmission Rates Among Patients With COVID-19 After Discharge From Acute Care Setting With Supplemental Oxygen
Source: JAMA Netw Open. 2021 Apr 1;4(4):e213990. doi: 10.1001/jamanetworkopen.2021.3990 (PMC8017465; doi:10.1001/jamanetworkopen.2021.3990)
Supplement: Supplement. — eAppendix 1. DHS Expected Practices: COVID19 Admission, Discharge, and Home O2 eAppendix 2. DHS Safer at Home – Home Oxygen Discharge Instructions [file jamanetwopen-e213990-s001.pdf]

Supplemental Online Content

Banerjee J, Canamar CP, Voyageur C, et al. Mortality and readmission rates among patients with COVID-19 after discharge from acute care setting with supplemental oxygen. *JAMA Netw Open*. 2021;4(4):e213990. doi:10.1001/jamanetworkopen.2021.3990

eAppendix 1. DHS Expected Practices: COVID19 Admission, Discharge, and Home O_2

eAppendix 2. DHS Safer at Home – Home Oxygen Dischharge Instructions

This supplemental material has been provided by the authors to give readers additional information about their work.

© 2021 Banerjee J et al. *JAMA Network Open*.

Health Services
LOS ANGELES COUNTY

DHS

Expected Practices Specialty: Inpatient and Emergency Department Services

Subject: COVID19 Admission, Discharge, and Home O_2 Criteria

Date: April 16, 2020

Purpose: To outline patient admission and discharge criteria, and selection criteria for initiation of home oxygen for patients, who are COVID-19 positive or COVID suspects.

Target Audience:
Inpatient and ED Practitioners.

Expected Practice:
Hospital Admissions:

- As per usual practice, only COVID-19+ patients meeting admissions criteria by InterQual should be admitted
- While physician discretion always applies, consider admission for COVID-19+ patients who
  - Are hemodynamically unstable (P>110, RR>22); or
  - Are persistently hypoxic despite supplemental oxygen (SaO2<94% while

This *Expected Practice* was developed by the DHS Covid Oversight Committee to fulfill the DHS mission to ensure access to high-quality, patient-centered, and cost-effective health care. Like the SPC Work Groups, the DHS COC, is guided by 1) real-life practice conditions at our facilities, 2) available clinical evidence, and 3) the principle that we must provide equitable care for the entire population that LA County DHS is responsible for, not just those that appear in front of us. It is recognized that in individual situations a provider’s clinical judgment may vary from this *Expected Practice*, but in such cases compelling documentation for the exception should be provided in the medical record.

© 2021 Banerjee J et al. *JAMA Network Open*.

on oxygen) or desaturate with exertion/ambulation despite supplemental oxygen; or

- Are demonstrating accessory muscle use while breathing or have an increased work of breathing despite nasal canula oxygen at 0-3 liters; or
- Who have other medical problems necessitating admission, aside from COVID-19+

<u>Discharge from ED or Inpatient Setting:</u>
While physician clinical judgment always applies, patients can be considered safe for discharge with respect to COVID-19 when:

© 2021 Banerjee J et al. *JAMA Network Open*.

- Oxygen requirements are 0-3L NC to keep SaO2 >/= 94% or improving clinical trajectory with SaO2 >/=92%, and without accessory muscle use; and
- No other medical issues necessitate continued acute inpatient care

To obtain home oxygen, do:

- Place order in ORCHID for Home Oxygen, including the rate (e.g., 2L NC/Min) and duration (e.g., 2 weeks)
- Record SaO2 on room air in BOTH RN Flow Sheet AND Physician Note
- Call your facility's DME Vender to arrange home O2 delivery and portable pulse oximeter, if available
- Call SW or UR for assistance
- If DME Vendor unavailable to deliver home oxygen, please follow ED Safer at Home Protocol for Home Oxygen
- Be sure to register the patient being discharged for a follow-up phone call to ensure patient remains clinically stable

- Other Symptoms:
  - Patients with fevers, myalgias, and cough are all OK to send home (just like patients with influenza) so long as patients are hemodynamically stable (see above) and can maintain an SaO2>92-94% while on 1-3L of supplemental oxygen

- Housing
  - Persons under investigation (PUI) and COVID-positive patients:
    - Need to self-isolate or go to isolation unit
      - Work with SW if homeless and need isolation unit
    - OK to go home and live with family/roommates IF they are also symptomatic
  - Provide patient with:
    - Isolation Order Summary from COVID-19 order set including link to Public Health Isolation Order and the
    - LA County Department of Public Health Home Isolation Instructions to patient

ED Safer at Home Protocol

Each hospital should establish a mechanism to accept oxygen tanks on consignment in the ED to allow for discharges on O2 after hours and on weekends/holidays. It is likely that no more than 5-10 oxygen tanks will be needed initially for consignment (based on initial experience at LAC+USC Medical Center).

- Each hospital shall have a process whereby stable PUIs or COVID-Positive individuals may be sent home with home oxygen

© 2021 Banerjee J et al. *JAMA Network Open*.

- This protocol entails:
    - ED-dispensed Home Oxygen, portable pulse oximeter (if available) and associated supplies
    - Facility-based RN and MD follow up phone call within 12-18 hrs
    - DME Vendor will follow up with patient within 24hrs to ensure equipment working

© 2021 Banerjee J et al. *JAMA Network Open*.

eAppendix 2. DHS SAFER AT HOME – HOME OXYGEN DISCHARGE INSTRUCTIONS

DHS SAFER AT HOME – HOME OXYGEN DISCHARGE INSTRUCTIONS

Thank you for choosing our Emergency Department/Hospital for your medical care. You have a virus that is causing difficulty breathing, but it is safe for you to go home with oxygen. Please remember to rest at home, do not go out except to get medical care, and stay away from other people until it is safe to stop self-isolation.

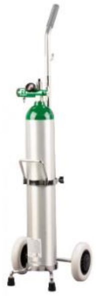

How Will I Get Oxygen?

You will get an oxygen tank from the hospital before you go home. Make sure to use the oxygen tank until you get home and the other machine is ready to use. Oxygen tanks do not need batteries or electricity.

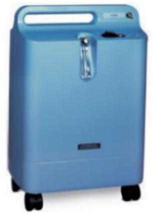

An oxygen company will deliver extra oxygen tanks and a machine called a "concentrator" to your home. The concentrator needs to be plugged into an electrical outlet. Use the oxygen tank until you can use the concentrator. Once you start using the concentrator, use the oxygen tanks only in an emergency, such as when you need to go back to the hospital.

The oxygen company may call you at the phone number you gave us. Call them if you do not hear from them within one hour of leaving the Emergency Department or Hospital.

Main oxygen companies* and contact numbers:

Olive View Medical Center – CalOx (323) 255-5175

Harbor-UCLA Medical Center – Supercare (800) 206-4880

LAC+USC Medical Center - CalOx (323) 255-5175

*These are the main companies used by the Department of Health Services (DHS), but you may get another company based on your health plan. Call your health plan if you are not sure which company is delivering your oxygen.

© 2021 Banerjee J et al. *JAMA Network Open*.

Check Your Symptoms at Home

You will also get a small machine called a "pulse oximeter" to check your oxygen level and heart rate at home. These numbers tell you how well your heart and lungs are working.

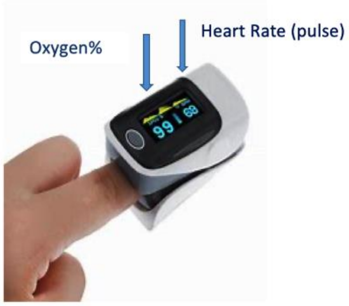

Check your oxygen level and symptoms every two hours while you're awake and record your numbers in the log. **If it becomes harder for you to breathe at any time, call 911 or go to the Emergency Department right away.** Call the oxygen company if you are having trouble with oxygen tanks or any other equipment.

Your oxygen level goal is 92%-94%:

- If you need 1-3 liters of oxygen per minute to get to 92-94%, you are SAFE.
- If you need 3-4 liters of oxygen per minute to get 92-94%, you are BORDERLINE.
- If you need more than 4 liters of oxygen per minute, you are UNSAFE. Go to the Emergency Department right away.

Oxygen Level Log:

Pulse Oxygen Recording					Any Symptoms?			
Date	Time	Oxygen Flow (L)	Oxygen Level (%)	Heart Rate (pulse)	Fever	Cough	Weakness	Chest pain

What if I Need Medical Advice?

Original 5/8/20 Updated 5/26/20

© 2021 Banerjee J et al. *JAMA Network Open*.

If you need medical advice, please call your doctor or the Department of Health Services (DHS) COVID-19 Nurse Advice Line at **1-844-804-0055**. Service hours are Monday – Sunday from 7AM-7PM.

How will I follow up about my symptoms?

You should have a follow-up phone visit with your doctor within 3 days after discharge. Call your doctor or health plan if you do not get a call about a follow-up visit. If you do not get a call and do not have a primary care provider or home clinic, call the Emergency Department or Hospital where you were seen.

© 2021 Banerjee J et al. *JAMA Network Open*.

DHS Emergency Departments:

Olive View Medical Center: (747) 210-4324

Harbor-UCLA Medical Center: (424) 306-5002

LAC+USC Medical Center: (323) 409-1000

DHS Hospitals:

Olive View Medical Center: (747) 210-3000

Harbor-UCLA Medical Center: (424) 306-4000

LAC+USC Medical Center: (323) 409-1000

© 2021 Banerjee J et al. *JAMA Network Open*.

eMaterials 2. DHS SAFER AT HOME – HOME OXYGEN DISCHARGE INSTRUCTIONS

© 2021 Banerjee J et al. *JAMA Network Open*.
